# CyclinD1 inhibits dicer and crucial miRNA expression by chromatin modification to promote the progression of intrahepatic cholangiocarcinoma

**DOI:** 10.1186/s13046-019-1415-5

**Published:** 2019-10-07

**Authors:** Yongqiang Qi, Da Wang, Wenhua Huang, Bing Wang, Di Huang, Fei Xiong, Xiaoping Chen, Yongjun Chen

**Affiliations:** 1 Department of Biliary-Pancreatic Surgery, Tongji Hospital, Tongji Medical College, Huazhong University of Science and Technology, Wuhan, Hubei Province China; 2 Department of Hepatic Surgery, Tongji Hospital, Tongji Medical College, Huazhong University of Science and Technology, Wuhan, Hubei Province China

**Keywords:** CyclinD1, Dicer, Methylation, Cholangiocarcinoma

## Abstract

**Background:**

CyclinD1 is crucial for cell cycling and can regulate the expression of Dicer, a crucial regulator of microRNA maturation. However, little is known on how CyclinD1 regulates Dicer and miRNA expression, and the progression of intrahepatic cholangiocarcinoma (ICC).

**Methods:**

The expression of CyclinD1 and Dicer in non-tumor cholangiocytes, ICC cells and tissues as well as their association with clinicopathological characteristics and survival were examined. The potential mechanisms by which CyclinD1 regulates Dicer and relative miRNA expression were determined by immunoprecipitation, ChIP sequence, BSP and luciferase reporter assays following induction of CyclinD1 over-expression or silencing and Dicer silencing. The impact of CyclinD1 and/or Dicer silencing on the growth of ICC was tested in vivo.

**Results:**

Up-regulated CyclinD1 was associated with down-regulated Dicer expression in ICC tissues and poorer overall survival in patients with ICC. CyclinD1 interacted with the nuclear H3K9me3 and SUV39H1 and bound to the Dicer promoter to increase its CpG island methylation in ICC cells. Functionally, CyclinD1 silencing inhibited the malignancy of ICC cells, which were mitigated partially by Dicer silencing in ICC cells. Dicer silencing down-regulated miR-1914-5p and miR-541-5p expression, which targeted and promoted CyclinD1 and CDK6 expression in ICC cells.

**Conclusions:**

Our findings uncover that CyclinD1 inhibits Dicer expression by chromatin modification to reduce miR-1914-5p/miR-541-5p expression, which positively-feedback enhances CyclinD1 and CDK6 expression and progression of ICC.

**Electronic supplementary material:**

The online version of this article (10.1186/s13046-019-1415-5) contains supplementary material, which is available to authorized users.

## Background

Intrahepatic cholangiocarcinoma (ICC) is the second most common primary liver malignancy. ICC comes from the bile ducts and its incidence is recently increasing [1, 2]. Currently, although several therapeutic strategies, such selective resection and intrahepatic arterial infusion of chemotherapy, are available, their efficacy is limited. Hence, understanding the molecular pathogenesis and discovery of new therapeutic targets are of significance in management of patients with ICC [3, 4].

Previous studies have shown that epigenetic modifications, such as the promoter hyper-methylation and dysregulated microRNA expression, have been associated with the development and progression of ICC [5, 6]. For example, 76% of patients have the hyper-methylation in the INK4A (p16) [7], 88% in the SOCS-3 [8], 69% in the RASSF1A [9], and 100% in the SEMA3B [10], leading to their down-regulated expression. Furthermore, the expression of a cluster of 38 miRNAs is dysregulated in ICC and some of them were associated with aberrant signaling (e.g., HGF/MET, IL-6/STAT-3) [6]. However, the factors contributing to dysregulated miRNA expression and epigenetic alterations during the development of ICC have not been clarified.

Dicer, a member of the RNase III endoribonuclease family, is crucial for the maturation of miRNA and siRNA [11, 12]. The Dicer and its regulated miRNAs are important to maintain the methylation status of the CpG islands in the promoter during the development of some types of malignancies [13–16]. Although miRNAs act as oncogenes or tumor suppressors during the development of cancers [17] a decrease in the levels of mature miRNA expression is detected in human cancers [18], suggesting that deficient expression of Dicer is associated with the development of malignant tumors, including ICC [19]. However, what transcription factors and co-activators control the Dicer expression during the development and progression of ICC has yet to be clear.

CyclinD1, one member of the highly conserved cyclin family, is encoded by the *CCND1* gene in humans and is an important regulator of cyclin-dependent kinases, such as CDK4 and CDK6 to control the cell cycle G1/S transition by inhibiting the cell cycle inhibitor expression [20–23]. The CyclinD1 over-expression due to the mutation, amplification and rearrangement of the *CCND1* gene is commonly detected in different types of malignant tumors [24–27]. In addition, CyclinD1 can bind to nuclear receptors to promote the proliferation of some types of cells and to histone acetylases and deacetylases to epigenetically regulate gene expression [28]. However, CyclinD1 may also act as a co-activator to control gene expression in a CDK-independent manner. For example, recent studies have shown that CyclinD1 induces Dicer expression in breast cancer by governing miRNA expression [29–31]. But whether and how CyclinD1 can regulate the Dicer and downstream miRNA expression in ICC have not been clarified.

In this study, we identified a new mechanism by which CyclinD1 inhibited Dicer expression by hyper-methylation of the Dicer promoter, which reduced miR-1914-5p and miR-541-5p expression that targeted CyclinD1 and CDK6 expression to promote the progression of ICC.

## Methods

### Antibodies and samples

Antibodies included rabbit anti-CyclinD1 (Abcam, Cambridge, MA, USA, ab6152), anti-H3K9me3 (Abcam, ab8898), anti-CDK6 (Abcam, ab108357), polyclonal anti-SUV39H1 (Proteintech, Wuhan, China, 10,574–1-AP), mouse monoclonal anti-Dicer (Abcam, ab14601), rabbit polyclonal anti-Dnmt1 (Santa Cruz Biotech, Dallas, TX, USA, sc-20,701), polyclonal anti-Dnmt3a (Santa Cruz Biotech, sc-20,703), polyclonal anti-Dnmt3b (Santa Cruz Biotech, sc-20,704), SUV39H1 (Proteintech, 10,574–1-AP), goat polyclonal anti-HP1α (Abcam, ab77256), rabbit polyclonal anti-Histone3 (Proteintech, 17,168–1-AP), mouse monoclonal anti-β-actin (BOSTER, Wuhan, China, BM0626), mouse monoclonal anti-GST (Proteintech, 66,001–1-Ig) and IgG from healthy animals (Beyotime, Shanghai, China, A7007).

Fourteen pairs of human ICC and adjacent non-tumor tissues were obtained from the Department of Biliary-Pancreatic Surgery, Tongji Hospital of Huazhong University of Science and Technology (HUST, Hubei, China). Written informed consent was obtained from individual patients and the experimental protocol was approved by the Ethics Committee of the Tongji Hospital.

### Cell culture and transfection

Human ICC HuccT-1, HCCC9810, RBE cells and cholangiocyte HIBEpic cells were cultured in RPMI 1640 supplemented with 10% fetal bovine serum (FBS) in a humidified incubator containing 5% CO_2_ at 37 °C. HEK293T cells were cultivated in 10% FBS DMEM/high glucose medium. The lentivirus for Dicer-specific saRNA, CyclinD1-specific siRNA and control lentivirus (LV-NC, short for LV-siR-negative control) were constructed and generated by Genechem (Shanghai, China). After extraction from HEK293T cells, the cDNA fragment for the targeted gene was amplified by PCR and then subcloned into the lentiviral vector pGC-LV containing tagged green fluorescent protein (GFP). The final lentiviral construct U6-MCS-Ubi-EGFP was verified by DNA sequencing. Following transfection, the lentivirus was generated in HEK293T cells. The different groups of cells were transduced with lentiviruses (0.3 ml, 10^7^ TU/ml) in complete medium containing polybrene for 24 h and cultured in fresh RPMI1640 medium for additional 48 h in the presence of puromycin (4 μg/ml) to generate stable cell lines.

### RNA extraction and quantitative real-time PCR (qRT-PCR)

Total RNA was extracted from the different groups of cells using Trizol reagent (Invitrogen) and each RNA sample (400 ng) was reversely transcribed into cDNA using a PrimeScript RT Reagent kit (Takara, Dalian, China), according the manufacturer’s instruction. The relative levels of targeted gene mRNA or miRNA transcripts to the control Histone3, U6 RNA, respectively, were determined by qRT-PCR using the SYBR Premix Ex Taq (Takara) kit and specific primers in the iQ5 quantitative PCR detection system (Bio-Rad, Richmond, CA, USA). The sequences of primers were forward 5′-TGTGATICCGAGGAATTGGA-3′ and reverse 5′-CACGGTICCCAGTCTAICCCA-3′ for Dicer (41 bp); forward 5′-CAGAGGCGGAGGAGAACAAA-3′ and reverse 5′-ATGGAGGGCGGATTGGAA-3′ for CyclinD1 (38 bp); forward 5′-cgcgAAAGGATTCTGCTGTCGGT-3′, and reverse 5′-ATCCAGTGCAGGGTCCGAGG-3′ for hsa-miR-541-5p (43 bp); forward 5′-CCCTGTGCCCGGCCC-3′ and reverse 5′-ATCCAGTGCAGGGTCCGAGG-3′ for hsa-miR-1914-5p (35 bp); forward 5′-GACTCATGACCACAGTCCATGC-3′ and reverse 5′-AGAGGCAGGGATGATGTTCTG-3′ for GAPDH (43 bp); forward 5′-CTCGCTTCGGCAGCACA-3′ and reverse 5′-ATCCAGTGCAGGGTCCGAGG-3′ for U6 RNA (37 bp). The data were analyzed using the IQ5 software.

### Western blotting

The different groups of cells were harvested and lyzed in lysis buffer containing protease inhibitors and phosphatase inhibitor (Roche), followed by centrifugation. After qualification and quantification, the cell lysates (50 μg) were separated by sodium dodecyl sulfate polyacrylamide gel electrophoresis (SDS-PAGE) on 12% gels and transferred onto polyvinylidene fluoride (PVDF) membrane (Millipore, Billerica, MA, USA). After blocked with 5% fat-free dry milk in TBST, the membranes were incubated with corresponding antibodies overnight at 4 °C and the bound antibodies were detected by horseradish peroxidase (HRP)-conjugated second antibodies and visualized using the enhanced chemiluminescence (ECL, Millipore). The relative levels of protein expression were determined by densitometric analysis using the Image J software.

### Immunofluorescence

The LV-NC-CyclinD1 and LV-siR-CyclinD1 HuccT-1 and HCCC9810 cells (10^5^ cells/well) were cultured on glass coverslips for 48 h, fixed in 4% paraformaldehyde and treated with 0.1% Triton x-100. The cells were stained with FITC-anti-CyclinD1, PE-anti-H3K9me3 or isotype controls. After being washed, the coverslips were mounted on the slides using the mounting medium containing DAPI. The fluorescent signals were detected under a fluorescent microscope.

### Co-immunoprecipitation

The potential interaction among the CyclinD1 with SUV39H1/H3K9me3/HP1α was determined by co-immunoprecipitation. Briefly, the cells were lyzed in IP lysis buffer (50 mM Tris-HCl, pH 7.4, 150 mM NaCl, 1 mM EDTA, 1% NP-40 and 10% Glycerin) containing protease inhibitors and phosphatase inhibitor (Roche). The cell lysate samples were incubated with 2.5 μg anti-CyclinD1/H3K9me3/HP1α antibodies in the presence of 20 μl Protein A + G Agarose beads (Beyotime) overnight at 4 °C with gentle shaking. After being washed, the bound proteins were eluted and they, together with cell lysates, were characterized by immunoblotting using anti-CyclinD1, anti-Dnmt3a, anti-Dnmt3b, anti-SUV39H1 and anti-H3K9me3.

### Immunohistochemistry

An ICC tissue microarray was purchased from Shanghai Outdo Biotech (Shanghai, China) and included 27 ICC tissues and 9 paracancerous non-tumor tissues. All samples were deparaffinized, rehydrated, and subjected to antigen retrieval. The sections were probed with anti-CyclinD1 (1:500) at 4 °C overnight and after being washed, the bound antibodies were detected with biotinylated second antibodies for 30 min at 37 °C, followed by visualizing with streptavidin-biotin-peroxidase and DAB. The sections were counterstained with hematoxylin. Immunohistochemical signals were scored semi-quantitatively, according to the percentage and intensity of positive-staining cells. 0: < 5% positive cells; 1: 5 to 24% positive cells; 2: 25 to 49% positive cells; 3: 50 to 74% positive cells and 4: ≥ 75% positive cells. Intensity was scored as 0 for absence of staining, 1 for weak, 2 for moderate, and 3 for strong staining. The final staining index was equal to intensity × positive rate and classified as absent, 0–1; mild, 2–4; moderate, 5–8; and strong, 9–12 respectively.

### Chromatin immunoprecipitation (ChIP)

Approximately, 10^7^ cells were fixed with 1% formaldehyde to crosslink endogenous proteins and DNA. The cells were sonicated and the DNA/protein complex was immunoprecipitated with primary antibodies and protein A/G agarose beads using IgG as a negative control. The precipitated DNA and input DNA were analyzed by qRT-PCR using specific primers. The sequences of primers were forward 5′-TGTGATCCAGAGGAATTGGA-3′ and reverse 5′-ACGGTCCACAGTCTACCACA-3′ for Dicer; forward 5′-CCCTCCTCCTCTTCCTCAATCT-3′ and reverse 5′-AACGGCGCACGCTGATT-3′ for β-actin.

### Bisulfite sequencing PCR (BSP)

The BSP primers were designed using online MethPrimer program (http://www.urogene.org/methprimer). The sequences of primers for BSP were BSP-DICER-F(P1): 5′-GATAGGTGTGAGGGATATTTTTTT-3′, BSP-DICER-R(P1): 5′-TACAACCTCTACCTCCTAAATTCA-3′;BSP-DICER-F(P2): 5′-AATTTAGGAGGTAGAGGTTGTAG-3′, BSP-DICER-R(P2): 5′-AACCCCAATCATACATATAAAA-3′; BSP-DICER-F(P3): 5′-TTATTGGTTATTTATTTGTTGGAAGTT-3′, BSP-DICER-R(P3): 5′-AAATTCAAATAATTCTCCTACCTCA-3′. The BSP reactions were performed in 25 μl reaction mixture of ddH_2_O, 10 × PCR buffer, dNTP mix, PCR primers, rTaq and bisulfite converted DNA samples. The PCR products were subcloned into pMD19-T plasmid. A total of 10 clones from each group were randomly selected and sequenced by Oebiotech (Shanghai, China).

### Cell proliferation assay

The different groups of cells (5 × 10^3^ cells/well) were cultured in 96-well plates in 10% FBS RPMI 1640 medium for 48 h. During the last 1-h culture, individual wells were added with Cell Counting Kit-8 (CCK-8) reagent (Dojindo, Tokyo, Japan). The cell proliferation was measured for the optical density (OD) at 450 nm using a microplate reader (Bio-Rad).

### Wound healing and transwell invasion assay

The different groups of ICC cells (1 × 10^6^ cells/well) were cultured up to 90% confluency and the monolayer of cells in individual wells were scratched with a sterile pipette tip (100 μl). The cells were continually cultured for 24 h and imaged at 0 and 24 h post scratching using a digital camera (Leica, Heerburg, Germany). The extent of wound healing was assessed by the distance of migration into the denuded area.

The different groups of ICC cells (1 × 10^4^ cells/well) were cultured in the upper chambers of the 24-well transwell plates that had been coated with Matrigel (BD Biosciences, San Jose, CA, USA). The bottom chambers were added with complete medium. After culture for 36 h, the cells at the top surface of the upper chambers were removed using a cotton swab. The invaded cells at the bottom surface of the upper chamber membrane were fixed in 4% paraformaldehyde, stained with 1% crystal violet and counted under a microscope in a blinded manner.

### Xenograft tumor assay in mice

Male BALB/C nude mice at 4 weeks of age were obtained from the Beijing HFK Bioscience, Beijing, China and housed in a pathogen-free facility. Individual mice were injected subcutaneously with 2 × 10^5^ HuccT-1 cells in 200 μl PBS. The dynamic growth of implanted tumors were monitored every three days using a caliper up to 18 days post inoculation and the tumor volumes were calculated by the formula: (0.5 × length×width)^2^. All mice were sacrificed at 18 days post inoculation. This study was approved by the Experimental Animal Ethics Committee of Tongji Medical College of Huazhong University of Science and Technology.

### Flow cytometry analysis of apoptosis

The cells (1 × 10^5^ cells/mL) were stained with Annexin V-FITC (Abcam) and propidium iodide (PI) in the dark. After being washed, the cells were analyzed by flow cytometry in a flow cytometer (BD Bioscience, San Jose, CA).

### Luciferase reporter assay

HEK293T cells (1 × 10^5^ cells/well) were cultured in 96-well plates up to 50% confluency and co-transfected in triplicate with 0.5 μg pDicer-GL3 (or control pGL3) and 0.1 μg pDNA-CyclinD1 (or control pDNA) and Renilla plasmid using lipofectamine 2000 (Invitrogen). Twenty-four hours after transfection, the luciferase activities in different groups of cells were measured using the Dual-Luciferase Reporter Assay System (Promega) on an illuminometer (Lumat LB9507, Berthold, Germany). The firefly luciferase acted as a reporter gene and Renilla luciferase as a normalized control for analysis.

To explore the regulatory role of miR-1914-5p or miR-541-5p on the CyclinD1 and CDK6 expression, the HEK293T cells at 50% confluency in 96-well plates were co-transfected with hsa-miR-1914-5p/541-5p or anti-miR-1914-5p/541-5p, along with reporter vectors using lipofectamine 2000 (Invitrogen). The firefly and Renilla luciferase activities were measured at 48 h post transfection.

### Statistical analysis

All data are expressed as the mean ± S.D. The difference between two independent samples was tested by two-tail Student’s t-test and the difference among the groups was determined by two-tail ANOVA using GraphPad Prism 5.0 (GraphPad Prism Software, San Diego, CA, USA). A *P*-value of < 0.05 was considered statistically significant.

## Results

### Up-regulated CyclinD1 is associated with down-regulated dicer expression in ICC tissues and related to poor prognosis in patients with ICC

**Fig. 1:**
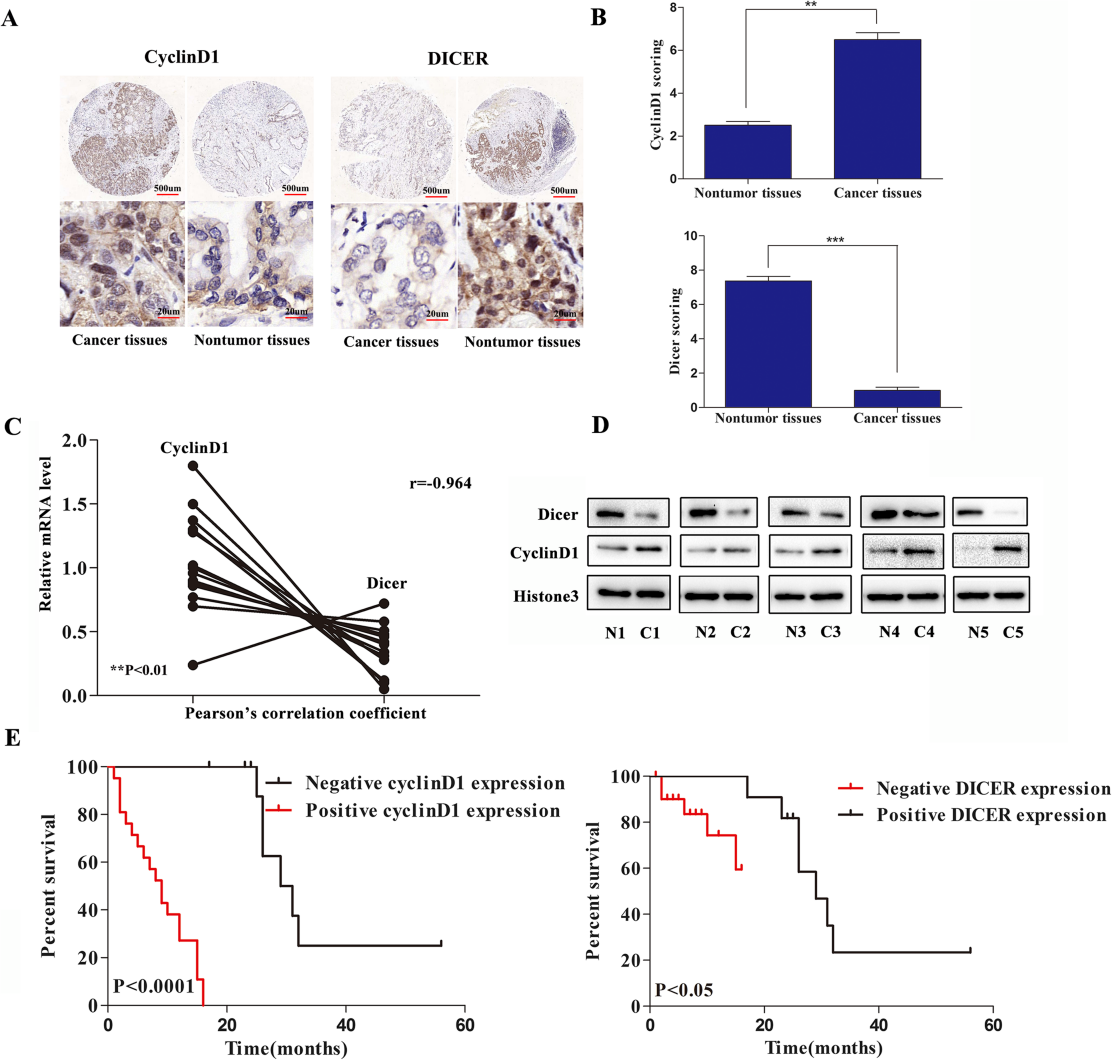
Up-regulated CyclinD1 is associated with down-regulated Dicer expression and poor survival in patients with ICC. **a**-**b** The levels of CyclinD1 and Dicer in 27 primary ICC and 9 adjacent non-tumor tissues were examined by IHC. Scale bar, 500 μm (upper panel, red line) and 20 μm (low panel, red line) respectively. **b** The CyclinD1 expression (upper panel) and Dicer expression (low panel) were examined by semi-quantitatively analyzed. **c** The levels of CyclinD1 and Dicer mRNA transcripts in 14 pairs of ICC and adjacent non-tumor tissues were determined by quantitative RT-PCR and the coefficient was examined by Pearson correlation (*r* = − 0.964, **p < 0.01). **d** Western blot analysis of the relative levels of CyclinD1 and Dicer to Histone3 expression in five pairs of ICC and adjacent non-tumor tissues. **e** Kaplan–Meier analysis of the overall survival of 33 ICC patients with high CyclinD1(*n* = 21) and lower Dicer (*n* = 22) expression vs. patients with lower CyclinD1 (*n* = 12) and higher Dicer (*n* = 11) expression. Data are representative images or expressed as individual mea values or the means ± SD of each group from three separate experiments. *p < 0.05, **p < 0.01, ***p < 0.001

To understand the potential relationship between CyclinD1 and Dicer during the development and progression of ICC, the levels of CyclinD1 and Dicer expression in 27 primary ICC and 9 adjacent non-tumor tissues were examined by immunohistochemitry (IHC). As shown in Fig. 1a, both CyclinD1 and Dicer expression were mainly localized in the nucleus of tissue cells. In comparison with that in the non-tumor biliary epithelial tissues, the CyclinD1 expression was up-regulated while the Dicer expression was down-regulated in the ICC tissues. Semi-quantitative analysis indicated that the levels of CyclinD1 expression in the ICC tissues were significantly higher than that of non-tumor tissues (6.50 ± 0.33 vs. 2.50 ± 0.20, *P* < 0.01) while the levels of Dicer expression in the ICC tissues were significantly lower than that in the non-tumor tissues (1.00 ± 0.19 vs. 7.38 ± 0.26, *p* < 0.001, Fig. 1b). Further qRT-PCR of 14 paired ICC and adjacent non-tumor tissue samples indicated that 13 out of 14 pairs of samples had higher levels of CyclinD1 expression and lower levels of Dicer expression with a Pearson correlation coefficient of − 0.964 (*P* < 0.01, Fig. 1c). Western blot analysis revealed that the relative levels of Dicer expression in the tumor samples were lower than that of the non-tumor tissues while the relative levels of CyclinD1 expression in the tumor tissues were higher than that in the non-tumor tissues (Fig. 1d). Collectively, the levels of CyclinD1 expression were inversely associated with Dicer expression in ICC tissues, which was different from Dicer expression in CCA [32].

Next, we investigated how the expression of CyclinD1 and Dicer was associated with clinicopathological features in 33 patients with ICC. We found that elevated expression of CyclinD1 was associated with pathologic grade and TNM stage (*P* < 0.001, *P* = 0.002, respectively), but not with age, sex, lymph node metastasis or distant metastasis (Table 1). In addition, the decreased levels of Dicer expression were associated significantly with the pathologic grade, TNM stage and distant metastasis (*P* < 0.001, *P* < 0.001 and *P* < 0.05, respectively) in this population. More importantly, stratification of patients with high (or positive) from low (or negative) expression of CyclinD1 or Dicer exhibited that the patients with higher CyclinD1 or lower Dicer expression had significantly shorter overall survival periods than those with lower levels of CyclinD1 or higher levels of Dicer expression in this population (Fig. 1e). Therefore, down-regulated Dicer expression were associated with up-regulated CyclinD1 expression in ICC tissues, relative to poor survival in patients with ICC.

**Table 1:** Association between CyclinD1/DICER expression and clinicopathologic characteristics

Characteristics	Numbers of Patients	CyclinD1		*P* value	DICER		*P* value
		Low expression	High expression		Low expression	High expression	
Age (years)				0.066			0.129
≤ 55	21	15	6		17	4	
> 55	12	8	4		6	6	
Gender				0.295			0.142
Male	19	10	9		12	7	
Female	14	8	6		10	4	
Pathology grade				< 0.001*			< 0.001
Low (I-II)	8	2	6		7	1	
High (III-IV)	25	9	16		22	3	
TNM stage				0.002*			< 0.001
I	4	2	2		4	0	
II	8	2	6		6	2	
III	11	4	7		10	1	
IV	10	3	7		8	2	
Lymph node metastasis				0.225			0.265
Negative	13	7	6		7	6	
Positive	20	11	9		13	7	
Distant metastasis				0.560			< 0.05
M0	16	8	8		9	7	
M1	17	10	7		13	4	

Low expression: IHC staining index≤6; High expression: IHC staining index> 6

### Knockdown of CyclinD1 enhances dicer expression in ICC cells

**Fig. 2:**
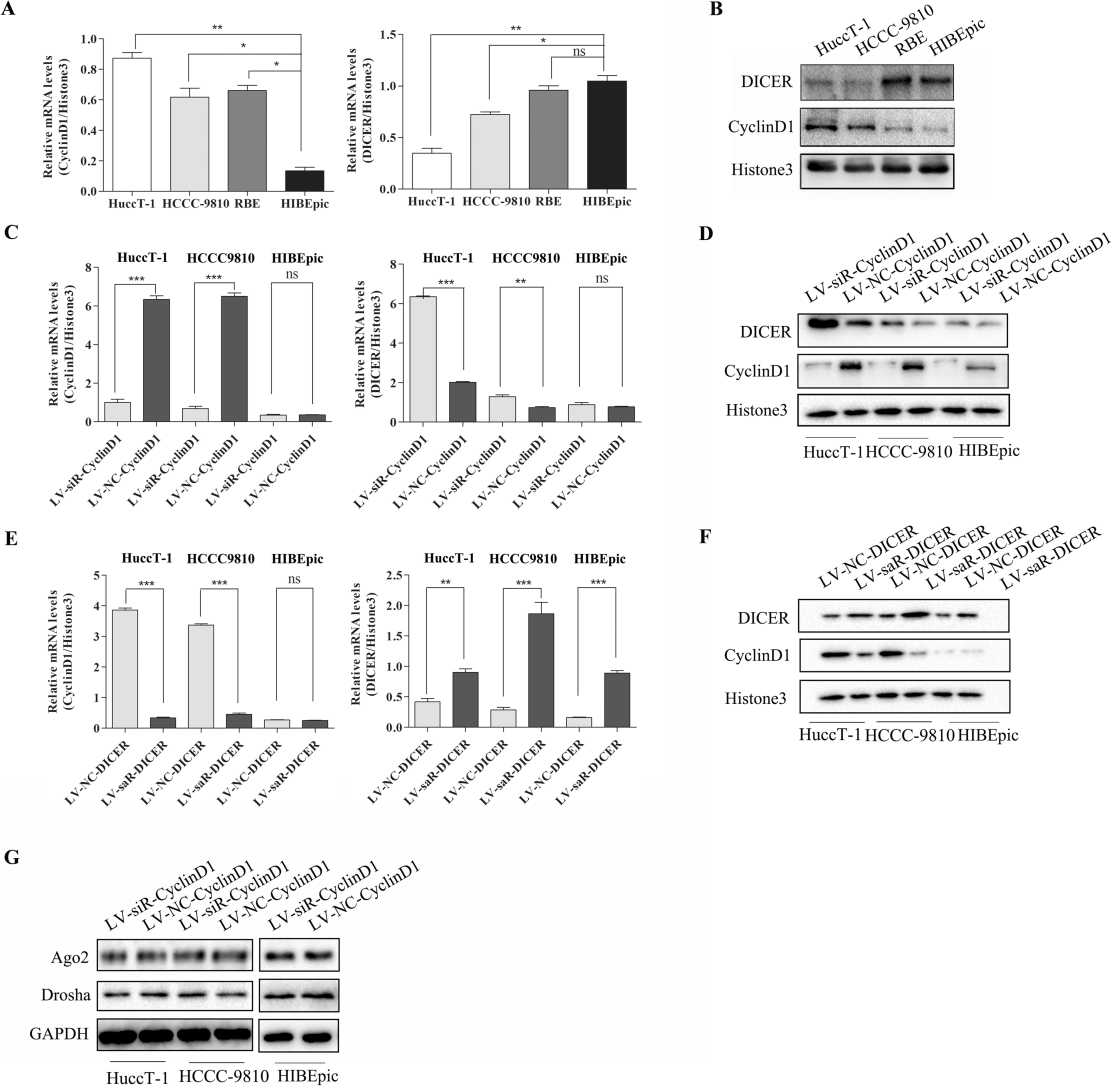
CyclinD1 silencing enhances Dicer expression while Dicer over-expression reduces CyclinD1 expression in ICC cells. **a**-**b** The relative levels of CyclinD1 and Dicer expression were determined respectively in ICC and non-tumor HIBEpic cells by quantitative RT-PCR and Western blot. **c**-**d** CyclinD1 silencing and related Dicer expression was demonstrated in HuccT-1, HCCC9810 and HIBEpic cells by quantitative RT-PCR and Western blot. **e**-**f** Dicer over-expression and CyclinD1 expression was demonstrated in HuccT-1, HCCC9810 and HIBEpic cells by quantitative RT-PCR and Western blot. **g** Western blot demonstrate that CyclinD1 silencing has no relation with expression two essential components of miRNA processing complex Ago2 and Drosha in HuccT-1, HCCC9810 and HIBEpic cells. Data are representative images or expressed as individual mea values or the means ± SD of each group from three separate experiments. *P < 0.05, **p < 0.01, ***p < 0.001

To understand the regulatory role of CyclinD1 in Dicer expression, we first tested the relative levels of CyclinD1 and CyclinD1 expression in ICC HuccT-1, HCCC9810 and RBE as well as non-tumor cholangiocyte HIBEpic cells by qRT-PCR and Western blot assays. As shown in Fig. 2a and b, the relative levels of CyclinD1 expression in ICC cells were significantly higher than that in the non-tumor HIBEpic cells. In contrast, Dicer expression in ICC cells was lower than that in HIBEpic cells.

Next, we employed lentivirus-mediated siRNA expression to silence CyclinD1 expression in HuccT-1, HCCC9810 and HIBEpic cells (Fig. 2c). We found that CyclinD1 silencing increased the relative levels of Dicer expression in both HuccT-1 and HCCC9810 cells, but not in HIBEpic cells (Fig. 2c and d). Similarly, we generated Dicer over-expressing HuccT-1, HCCC9810 and HIBEpic cells using saRNA technique (Fig. 2e). Interestingly, induction of Dicer over-expression reduced the levels of CyclinD1 expression in HuccT-1 and HCCC9810 cells, but not in HIBEpic cells, suggesting that Dicer may indirectly inhibit CyclinD1 expression in ICC cells (Fig. 2e and f).

Furthermore, CyclinD1 silencing increased Dicer expression, but did not affect the expression of Ago2 and Drosha in the silencing complex (Fig. 2g). Such data suggest that CyclinD1 may preferably regulate the expression of Dicer in ICC cells. Dicer, a RNase III endoribonuclease, can cleave long double-stranded RNA (dsRNA) or stem-loop-stem structured pre-miRNA to form mature miRNAs in the cytoplasm. Interestingly, we found that down-regulated Dicer expression was mainly located in the nucleus of ICC cells (Fig. 1a-b, Fig. 2b and Additional file 1: Figure S1A-C). Accordingly, we further explored whether Dicer participated in the alterations in the nucleolar structure. As shown in Additional file 1: Figure S1A-C, Dicer over-expression increased the levels of cytoplasmic and nuclear Dicer in HuccT-1 and HCCC9810 cells, particularly in the cytoplasm. However, inhibition of Dicer expression apparently altered the nucleolar structure of ICC cells. In contrast, altered Dicer expression did not change the distribution of Dicer in the cytoplasm and nucleus of HIBEpic cells. Our data indicated that CyclinD1 inhibited Dicer expression in ICC cells in a CDK-independent manner.

### CyclinD1 expression is predominantly co-localized in the nuclear with HP1α/H3K9me3/SUV39H1/Dnmt complex in ICC cells

**Fig. 3:**
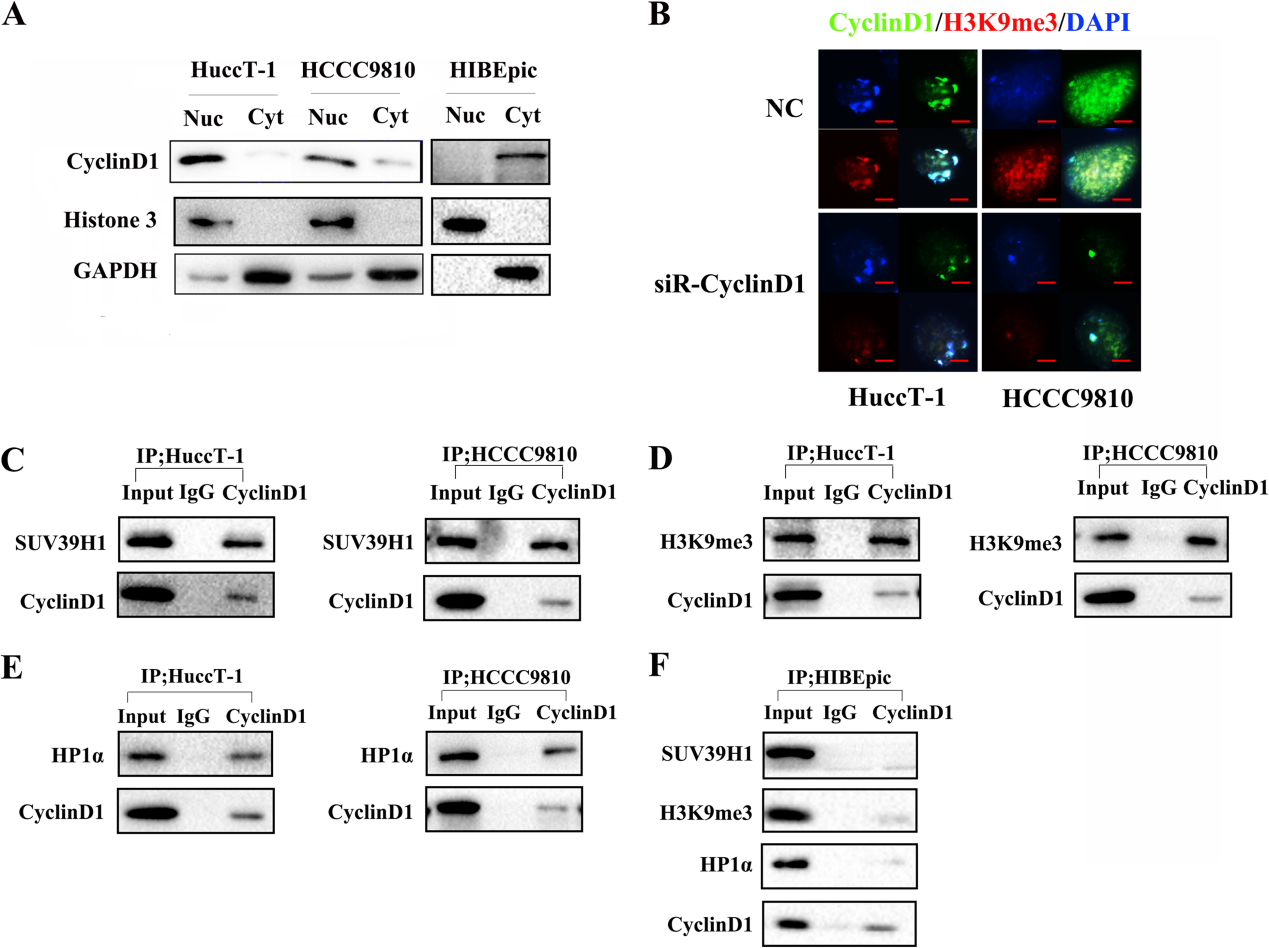
CyclinD1 and H3K9me3 are collocated in the nucleus and form a complex with SUV39H1, HP1α and DNMTs in ICC cells. **a** Western blot analysis reveals that CyclinD1 and H3K9me3 are mainly expressed in the nucleus in HuccT-1 and HCCC9810 cells. **b** Immunofluorescence (IF) analysis of the nuclear co-localization of CyclinD1 with H3K9me3 in HuccT-1 and HCCC9810 cells (400×, magnification). **c**-**f** Anti-CyclinD1 immunoprecipitates endogenously with SUV39H1, H3K9me3 and HP1α from HuccT-1, HCCC9810 and HIBEpic cells

A previous study has reported that CyclinD1 may regulate Dicer expression [31] and CyclinD1 can bind to histone acetylases and deacetylases to regulate gene expression [28]. However, the precise mechanisms underlying the action of CyclinD1 in regulating Dicer expression during the development of ICC have not been clarified. To understand the regulatory role of CyclinD1 in Dicer expression, we characterized the relative levels of CyclinD1 in the cytoplasm and nucleus, and found that CyclinD1, similar to H3K9me3, was predominantly located in the nucleus of HuccT-1 and HCCC9810 cells, but in the cytoplasm of HIBEpic cells (Fig. 3a). Following stained with FITC-anti-CyclinD1, PE-anti-H3K9me3 and DAPI, we found that CyclinD1 and H3K9me3 were co-localized mainly in the nuclei of HuccT-1 and HCCC9810 cells, but significantly reduced fluorescent signals were detected in the CyclinD1-silenced HuccT-1 and HCCC9810 cells (Fig. 3b). Given that the H3K9me3/SUV39H1/HP1α/Dnmts complex is crucial for chromatin modifications of gene expression in hepatocarcinoma (HCC) [33], our data suggest that CyclinD1 may interact with epigenetic regulators, such as H3K9me3, to inhibit Dicer expression in ICC cells.

To test the hypothesis, we performed co-immunoprecipitation (Co-IP) and found that anti-CyclinD1 precipitated histone methyltransferase Suv39H1 and H3K9me3 in HuccT-1 and HCCC9810 cells (Fig. 3c and d). Furthermore, anti-CyclinD1 also precipitated heterochromatin protein 1α (HP1α), which is crucial for the hetorochromatin formation (Fig. 3e). Moreover, anti-HP1α also precipitated Suv39H1, DNA methyltransferase 3A and 3B in both HuccT-1 and HCCC9810 cells (Additional file 1: Figure S1D), but not detected in HIBEpic cells (Fig. 3f). Such data indicated that CyclinD1 participated in the HP1α/H3K9me3/SUV39H1/Dnmt complex to inhibit Dicer expression by chromatin modifications in ICC cells.

### CyclinD1 promotes the DICER promoter methylation in ICC cells

To determine the molecular mechanisms by which CyclinD1 inhibited the Dicer expression, we selected eight of the most differentially methylated genes between the LV-siR-CylcinD1 and LV-NC HuccT-1 cells, and they included four hypermethylated genes and four hypomethylated genes (Fig. 3a). A previous report reveals that CyclinD1 specially binds to the P2 region (− 725~ − 655 bp) of the DICER promoter in breast cancer [31]. Bioinformatics exhibited that there were 24 CpG islands in the upstream of Dicer transcription start site, including the CpG island-enriched region (− 1235 ~ − 931 bp) and the P2 region.

**Fig. 4:**
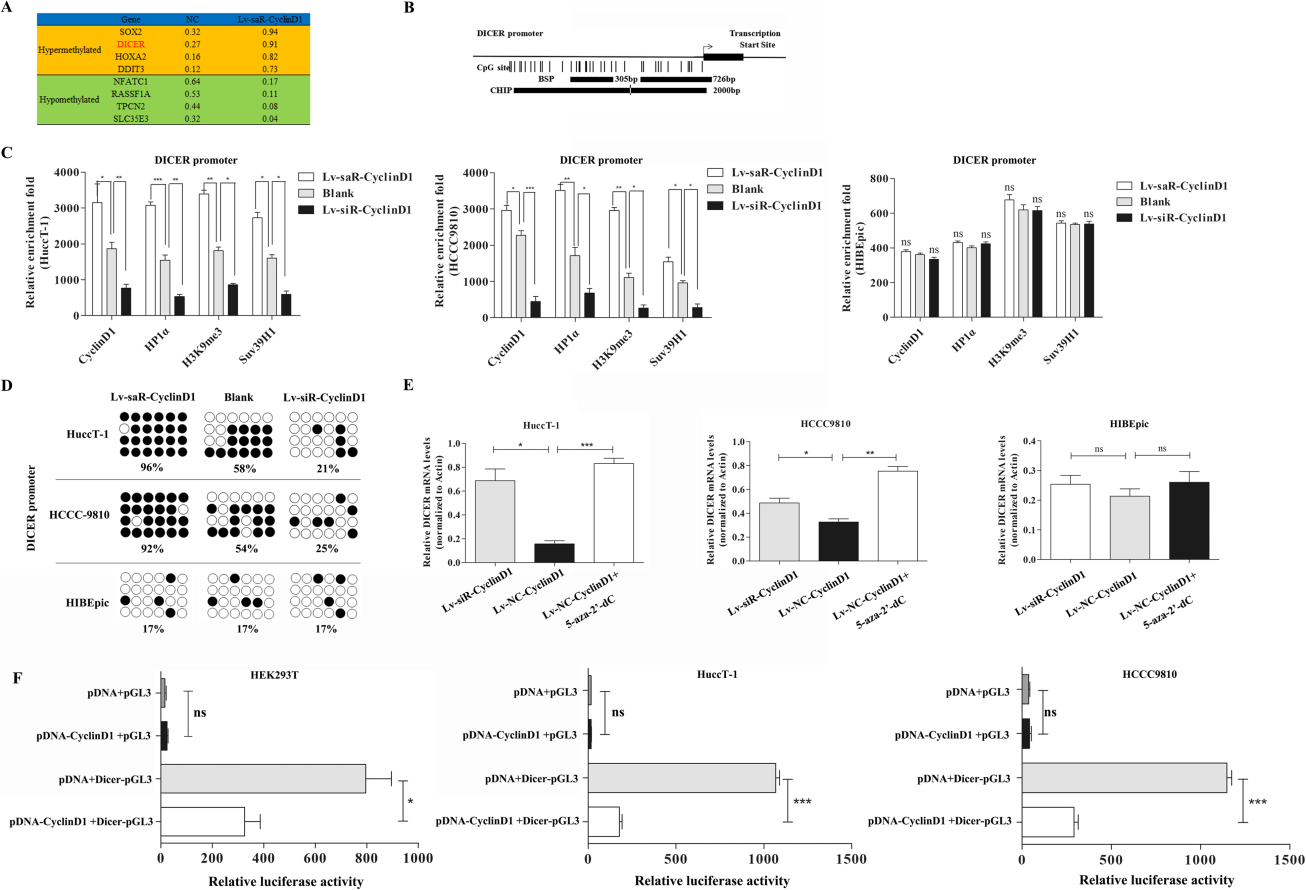
CyclinD1 binds to the Dicer promoter and increases the CpG island methylation to reduce Dicer expression in ICC and HIBEpic cells. **a** Listing of average DNA methylation rate of eight most differentially methylated genes between LV-siR-CyclinD1 and LV-NC, including four hypermethylated genes and four hypomethylated genes. **b** Graphic model of the CpG islands in the Dicer promoter. (**c**) ChIP analysis of CyclinD1, HP1α, H3K9me3 and SUV39H1 enrichment in the Dicer promoter region. IgG served as a negative control. Relative enrichment fold = [%(ChIP/Input)]/[%(IgG/Input)]. **d** BSP analysis of the CpG island methylation rate. Each row represents a single sequence, and every dot represents one CpG site. White and black dots represent unmethylated and methylated CpG islands, respectively. **e** Quantitative RT-PCR analysis of the relative levels of Dicer to β-actin mRNA transcripts in LV-siR-CyclinD1, LV-NC-CyclinD1 and LV-NC-CyclinD1 + 5-aza-dC groups. **f** Dual luciferase assays demonstrate that CyclinD1 attenuates the Dicer promoter-controlled luciferase activity in HEK293T and ICC cells (pDNA: empty plasmid vector; pGL3: empty pGL3 plasmid vector; pDNA-CyclinD1: pcDNA-CyclinD1 plasmid; Dicer-pGL3: The Dicer promoter inserted in the pGL3 plasmid). Data are expressed as individual mea values or the means ± SD of each group from three separate experiments. *P < 0.05, **p < 0.01, ***p < 0.001

Further ChIP and BSP assays indicated that compared with that in the control, CyclinD1 over-expression increased the relative levels of HP1α, H3K9me3 and SUV39H1 expression while CyclinD1 silencing significantly reduced their expression in both HuccT-1 and HCCC9810 cells, but not in HIBEpic cells (Fig. 4c). The BSP assay indicated that the percentages of methylated CpG in the control HuccT-1 and HCCC9810 cells were significantly lower than that in the Lv-saR-CyclinD1 cells (58% vs. 96%; 54% vs. 92%, *p* < 0.05), but significantly higher than that of the Lv-siR-CyclinD1 cells (58% vs. 21, 54% vs. 25%, *p* < 0.05, Fig. 4d). However, there was no significant change in the percentages of CpG methylation in the HIBEpic cells regardless of altered CyclinD1 expression (17% vs. 17% vs. 17%). Subsequently, CyclinD1 silencing or treatment with 5-Azacitidine (5-aza-2′-deoxycytidine) to induce demethylation significantly increased the relative levels of Dicer mRNA transcripts, relative to that of Lv-NC-CyclinD1 HuccT-1 and HCCC9810 cells, but not in HIBEpic cells (Fig. 4e).

We next determined how CyclinD1 regulated the Dicer promoter-controlled luciferase expression in HEK293T cells by dual luciferase assay. Following co-transfection, we found that induction of CyclinD1 over-expression significantly reduced the DICER promoter-controlled luciferase activity in HEK293T, HuccT-1 and HCCC9810 cells (Fig. 4f).

Because CyclinD1 inhibited Dicer expression by chromatin modifications, we further performed ChIP assays to determine whether altered CyclinD1 expression could also modulate the HP1α, H3K9me3 and SUV39H1 expression in the SOX2 and HOXA2 promoters in ICC and non-tumor HIBEpic cells. As shown in Additional file 2: Figure S2, CyclinD1 over-expression increased the relative levels of HP1α, H3K9me3 and SUV39H1 expression while CyclinD1 silencing significantly reduced their expression in the SOX2 and HOXA2 promoters of both HuccT-1 and HCCC9810 cells, but not in HIBEpic cells. Collectively, our data indicated that CyclinD1 was recruited into the H3K9me3/SUV39H1/HP1α/Dnmts complex in the DICER promoter to increase its CpG methylation and reduce Dicer expression in ICC cells.

### CyclinD1 silencing inhibits the proliferation and invasion of ICC cells, which are mitigated by dicer silencing

**Fig. 5:**
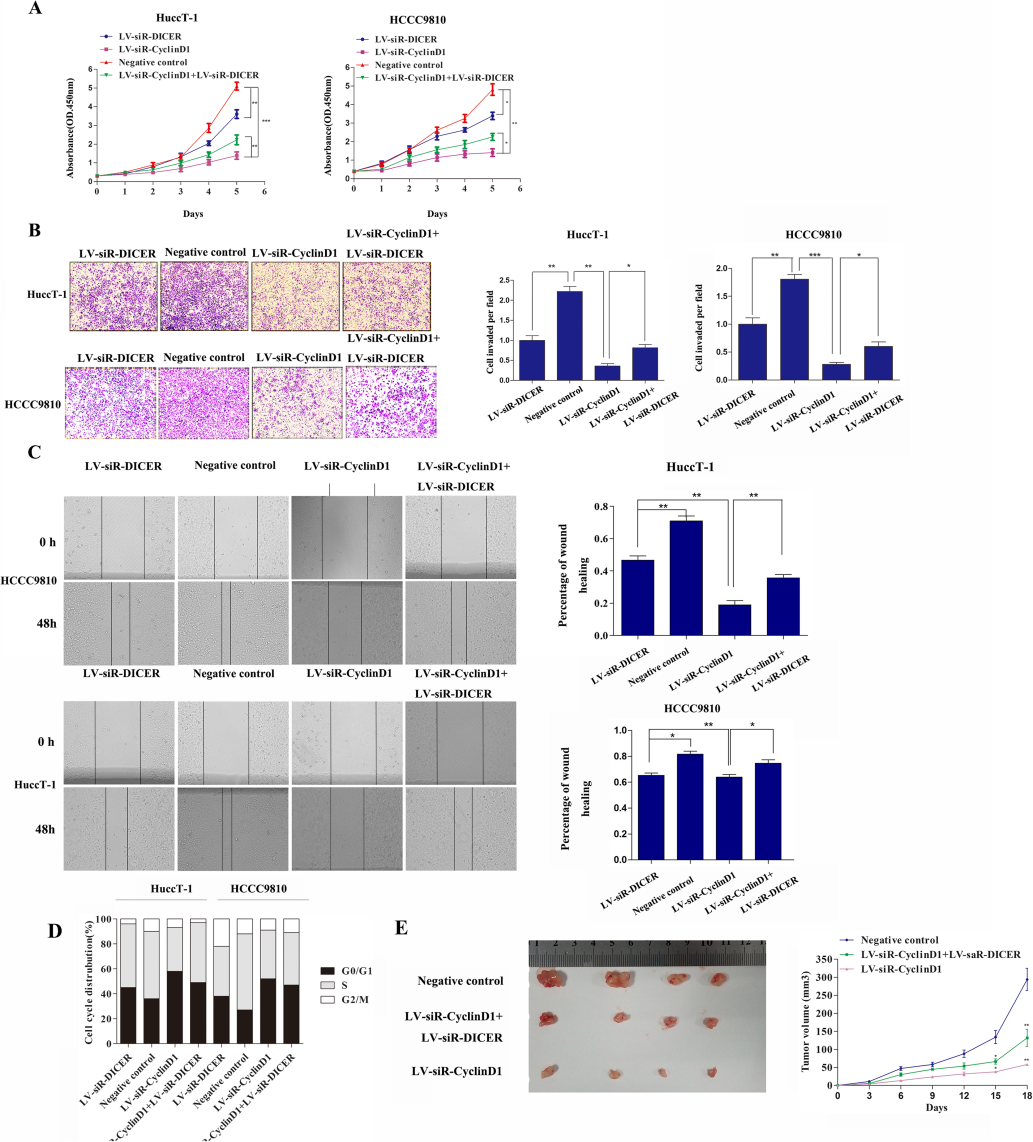
The effect of CyclinD1 and/or Dicer silencing on the proliferation and invasion of ICC cells. HuccT-1 and HCCC9810 cells were transduced with Lv-NC (negative control), Lv-siR-CyclinD1 and/or Lv-siR-Dicer to establish stable silencing cells. The proliferation, invasion, wound healing and cell cycling were determined. The growth of implanted negative control, Lv-siR-CyclinD1 and Lv-siR-CyclinD1/lv-siR-dicer HuccT-1 tumors in BALB/c nude mice was monitored. **a** The dynamic proliferation of ICC cells. **b** The invasion of ICC cells (200×, magnification). **c** The wound healing of ICC cells. **d** The distribution of cell cycle status of ICC cells. **e** The growth of implanted HuccT-1 tumors in mice (*N* = 4 per group). Data are representative images or expressed as individual mea values or the means ± SD of each group from three separate experiments. *P < 0.05, **p < 0.01, ***p < 0.001

Because CyclinD1 down-regulated Dicer expression, we investigated the impact of CyclinD1 and/or Dicer silencing on the proliferation and invasion of ICC cells. In comparison with that of the control, CyclinD1 silencing significantly inhibited the proliferation of HuccT-1 and HCCC9810 cells, which were significantly mitigated by Dicer co-silencing in both types of cells (Fig. 5a). In contrast, Dicer silencing alone only moderately reduced the proliferation of both types of cells. A similar pattern of data among the different groups of cells was observed from wound healing and transwell invasion assays (Fig. 5b and c). Moreover, CyclinD1 silencing induced cell cycling arrest at G0/G1 phase in both HuccT-1 and HCCC9810 cells, which were partially reduced by Dicer co-silencing (Fig. 5d). However, Dicer silencing alone only moderately increased the percentages of cells at G0/G1 phase in both HuccT-1 and HCCC9810 cells.

We further tested the effect of CyclinD1 and Dicer silencing on the growth of implanted HuccT-1 tumors. Individual BALB/C nude mice were implanted subcutaneously with HuccT-1 cells that had been transduced with the control lentivirus, Lv-siR-CyclinD1 alone or combined Lv-siR-CyclinD1 and Lv-siR-Dicer. The dynamic growth of implanted tumors in different groups of mice was monitored every three days up to 18 days post inoculation (Fig. 5e). Clearly, CyclinD1 silencing inhibited the growth of implanted HuccT-1 tumors in mice while CyclinD1 and Dicer co-silencing only moderately retarded the growth of implanted HuccT-1 tumors in mice. These data demonstrated that CyclinD1 silencing inhibited the proliferation and invasion of ICC cells, which were mitigated by Dicer silencing in vitro and in vivo*.*

### Dicer silencing down-regulates miRNA expression that promotes CyclinD1 expression in ICC cells

**Fig. 6:**
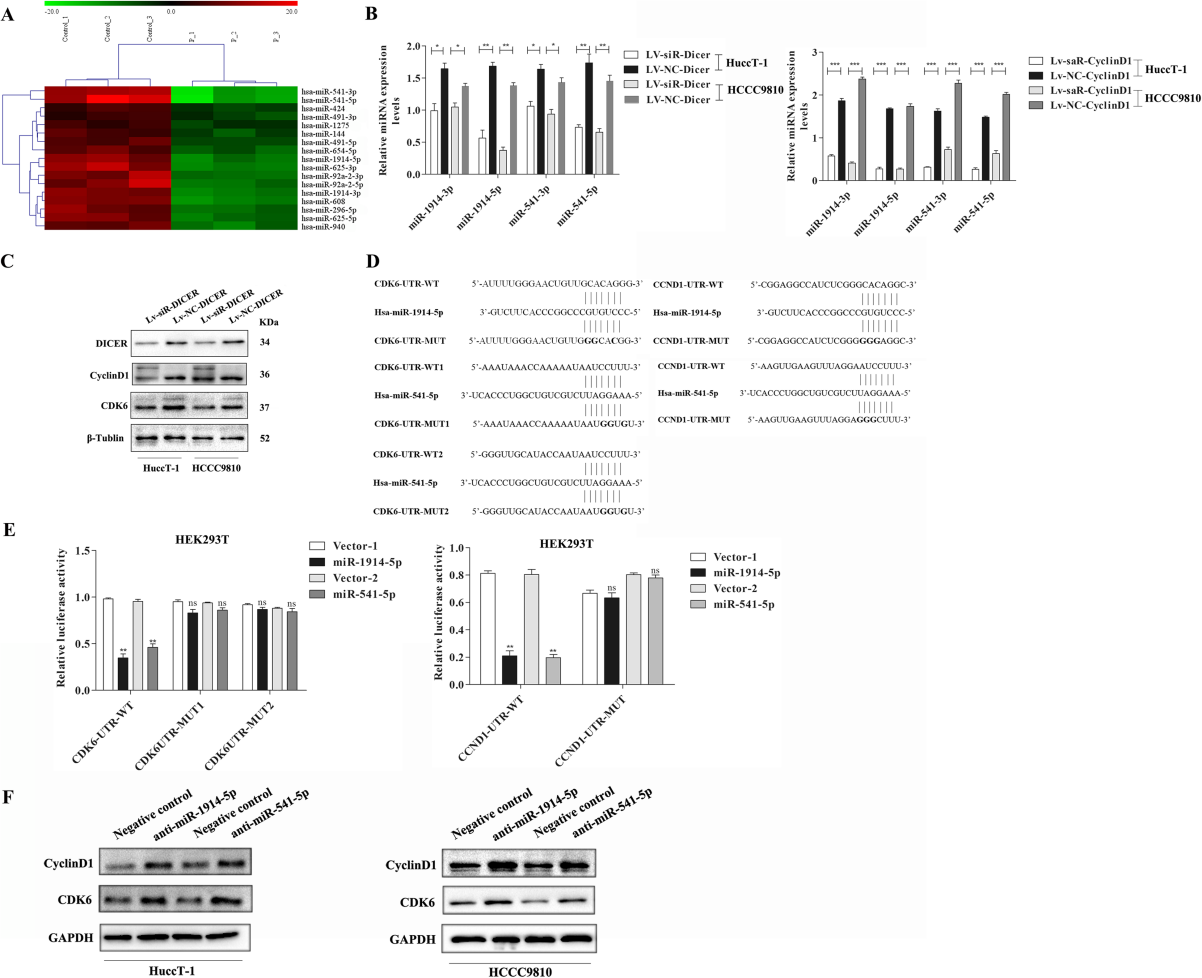
Dicer silencing reduces miR-1914-3p/5p and miR-541-3p/5p expression, leading to increased levels of CyclinD1 and CDK6 expression in ICC cells. **a** Microarray analysis of 17 miRNAs in ICC and control cells. **b** Quantitative RT-PCR analysis of miR-1914-3p/5p and miR-541-3p/5p expression in LV-siR-Dicer or Lv-saR-CyclinD1 and control of HuccT-1 and HCCC9810 cells. **c** Western blot analysis of Dicer/CDK6/CyclinD1 expression in in LV-siR-Dicer and control HuccT-1 and HCCC9810 cells. **d** Schematic illustration of the potential biding motifs for miR-1914-5p and miR-541-5p in the 3′-UTR of CDK6 and CyclinD1 and their mutants. **e** miR-1914-5p and miR-541-5p reduce the CyclinD1 and CDK6 3’UTR-regulated luciferase activity in HEK239T cells. **f** Western blot analysis reveals that knockdown of endogenous miR-1914-5p or miR-541-5p increases CyclinD1 and CDK6 expression in HuccT-1 and HCCC9810 cells. Data are representative images or expressed as individual mea values or the means ± SD of each group from three separate experiments. *P < 0.05, **p < 0.01, ***p < 0.001

Dicer is crucial for the maturation of miRNAs that target the 3’untranslated region (3’UTR) of mRNA to inhibit protein translation. Given that Dicer silencing mitigated the CyclinD1 silencing-decreased proliferation and invasion of ICC cells (Fig. 5a-d) we hypothesized that Dicer silencing might alter the levels of miRNA expression that targeted the CyclinD1 and/or its downstream molecule expression in ICC cells. To address it, HuccT-1, HCCC9810 and RBE cells were transfected with control Lv or Lv-shR-Dicer and the levels of miRNA expression were analyzed by miRNA microarray (Fig. 6a). In comparison with the control, there were 17 down-regulated miRNAs in the Dicer-silenced ICC cells. Further qRT-PCR demonstrated that Dicer silencing significantly reduced the relative levels of miR-1914-3p, miR-1914-5p, miR-541-3p and miR-541-5p expression in HuccT-1 and HCCC9810 cells (Fig. 6b). Similarly, CyclinD1 over-expression significantly reduced the relative levels of miR-1914-3p, miR-1914-5p, miR-541-3p and miR-541-5p expression in HuccT-1 and HCCC9810 cells.

Given that CyclinD1 can interact with CDK6 to promote the G1/S transition during the cell cycling [22], we explored whether Dicer silencing could form a positive feedback to up-regulate CyclinD1 and CDK6 expression by down-regulated miRNA expression. We first tested how Dicer silencing affected the levels of CyclinD1 and CDK6 expression in both of HuccT-1 and HCCC9810 cells by Western blot. We found that Dicer silencing did increase the relative levels of CyclinD1 and CDK6 expression in both types of cells (Fig. 6c). To understand how Dicer silencing affected the CyclinD1 and CDK6 expression, we employed bioinformatics and found that the 3’UTR of the CDK6 and CyclinD1 contained the motifs for the binding of miR-1914-5p and miR-541-5p (Fig. 6d). Subsequently, the regulatory effects of miR-1914-5p or miR-541-5p on the CyclinD1 and CDK6 expression were determined by dual luciferase assays. We found that transfection with miR-1914-5p or miR-541-5p significantly reduced the wild-type, but not the mutant CDK6- or CyclinD1 3’UTR-regulated luciferase expression in HEK293T cells (Fig. 6e). Finally, Western blot demonstrated that transfection with miR-1914-5p- or miR-541-5p-specific siRNA to neutralize the endogenous miR-1914-5p or miR-541-5p obviously increased the relative levels of CyclinD1 and CDK6 expression in both HuccT-1 and HCCC9810 cells (Fig. 6f). Therefore, Dicer silencing reduced the expression of critical miRNAs, leading to up-regulated CyclinD1 and CDK6 expression, promoting the cell cycling and ICC progression.

## Discussion

**Fig. 7:**
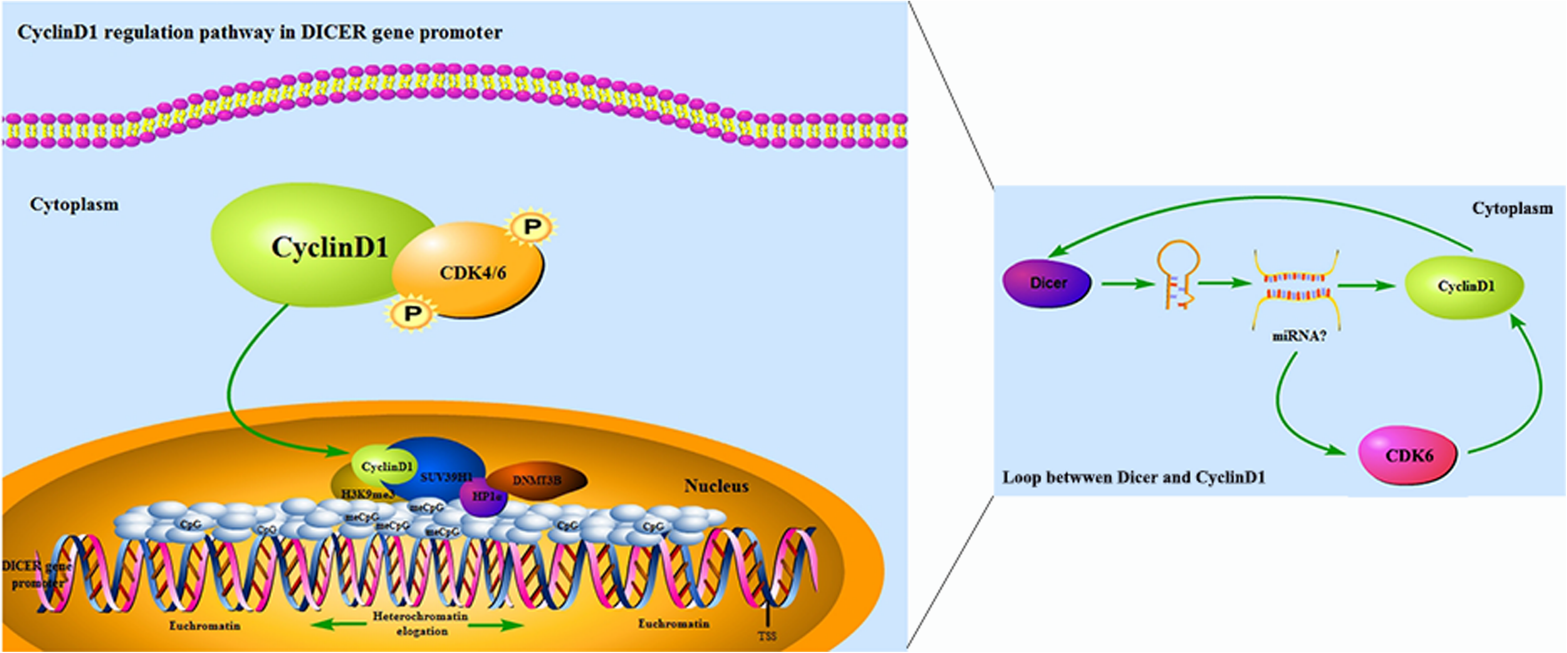
Schematic illustration of the role of CyclinD1 to regulate Dicer promoter in ICC. In nucleus, CyclinD1 recruits SUV39H1/H3K9me3/HP1α/DNMTs complex to promote Dicer promoter de novo methylation, and results in euchromosome transforming into heterochromatin, which represses Dicer transcription. Then the Dicer-induced miRNAs form a positive loop to participate in CyclinD1 regulation via CDK6/CyclinD1 signaling pathway

Dicer, as one kind of endonuclease protein, regulates the miRNA and siRNA maturation, and indirectly down-regulates the expression of related genes in a dose dependent manner [34]. Previous studies have shown that Dicer can regulate many biological function, including the CpG methylation, heterochromatin formation, cell cycling [13, 15, 35, 36]. Our previous study has shown that Dicer regulates the development and progression of cholangocarcinoma (CCA). Interestingly, a recent study indicates that CyclinD1 positively regulates Dicer expression in breast cancer cells [31]. In this study, we explored the role of Dicer and CyclinD1 in the development and progression of ICC (Fig. 7). We found that Dicer and CyclinD1 were expressed predominantly in the nuclei of ICC cells. Down-regulated Dicer expression was associated with up-regulated CyclinD1 expression in ICC tissues and with pathologic grade, TNM stage and poor survival of ICC patients. These suggest that the regulatory effect of CyclinD1 on Dicer expression may vary in different types of tumors. The significant association with a poor survival suggests that lower Dicer and higher CyclinD1 expression may be new biomarkers for prognosis in ICC patients.

To understand the regulatory role of CyclinD1 in Dicer expression, we characterized the relative levels of CyclinD1 expression in ICC and non-tumor HIBEpic cells and found that the levels of CyclinD1 expression in ICC cells were significantly higher than that in the non-tumor HIBEpic cells. Furthermore, CyclinD1 silencing enhanced Dicer expression while Dicer over-expression decreased CyclinD1 expression in ICC HuccT-1 and HCCC9810 cells, but not in HIBEpic cells. Interestingly, CyclinD1 was localized in the H3K9me3/SUV39H1/HP1α/Dnmts complex of HuccT-1 and HCCC9810 cells. Evidentially, the CyclinD1 and H3K9me3 expression were co-localized in the nuclei of HuccT-1 and HCCC9810 cells and anti-CyclinD1, anti-H3K9me3 or anti-HP1α effectively co-immunoprecipitated the H3K9me3, SUV39H1, Dnmt3B, and HP1α in HuccT-1 and HCCC9810 cells. Further ChIP assays revealed that CyclinD1 bound to the P2 region of the Dicer promoter and CyclinD1 over-expression significantly increased the methylation of CpG islands in the Dicer promoter, while CyclinD1 silencing had opposite effects in HuccT-1 and HCCC9810 cells. In addition, CyclinD1 silencing or treatment with 5-Azacitidine to induce demethylation significantly increased the relative levels of Dicer mRNA transcripts in HuccT-1 and HCCC9810 cells. Moreover, CyclinD1 over-expression significantly mitigated the Dicer promoter-controlled luciferase activity in HEK293T cells. Such data indicated that CyclinD1 bound to the Dicer promoter and recruited the H3K9me3, SUV39H1, Dnmt3B, and HP1α to promote the heterochromatin elongation and CpG island methylation, inhibiting Dicer expression in ICC cells [37]. To the best of our knowledge, this was the first evidence to demonstrate that CyclinD1 was a negative regulator of Dicer expression during the development and progression. These findings may provide new insights into the pathogenesis of ICC.

CyclinD1 can interact with CDK4 and CDK6, and is crucial for G1/S transition during cell cyclin process. Actually, we found that CyclinD1 silencing significantly attenuated the proliferation, wound healing and invasion of HuccT-1 and HCCC9810 cells in vitro, accompanied by inducing their cell cycle arrest at G0/G1 phase. Such inhibitory effects of CyclinD1 silencing were mitigated by Dicer co-silencing while Dicer silencing alone only moderately inhibited the proliferation, wound healing and invasion of HuccT-1 and HCCC9810 cells. In addition, CyclinD1 silencing significantly retarded the growth of implanted Hucc-T1 tumors in vivo, which was also mitigated by Dicer co-silencing. Such data suggest that Dicer silencing may promote the expression of oncogenic proteins, leading to progression of ICC. Given that Dicer is crucial for maturation of miRNAs we performed miRNA array and found that Dicer silencing significantly decreased miR-1914-3p, miR-1914-5p, miR-541-3p and miR-541-5p, but increased CyclinD1, CDK4 and CDK6 expression in HuccT-1 and HCCC9810 cells. Furthermore, we found that the 3UTR of CyclinD1 and CDK6 mRNAs contained the motifs for miR-1914-5p and miR-541-5p binding and transfection with miR-1914-5p or miR-541-5p significantly decreased the CyclinD1 and CDK6 3UTR-, but not their mutants-regulated luciferase activity. In addition, inhibition of endogenous miR-1914-5p or miR-541-5p significantly increased CyclinD1 and CDK6 expression in HuccT-1 and HCCC9810 cells. Such data indicated that Dicer silencing reduced miR-1914-5p and miR-541-5p expression, which formed a feedback loop to up-regulate CyclinD1 and CDK6 expression and promote the progression of ICC.

In summary, our data indicated that down-regulated Dicer was associated with up-regulated CyclinD1 expression in ICC tissues and poor survival in patients with ICC. CyclinD1 bound to the Dicer promoter and interacted with the HP1α/H3K9me3/SUV39H1/Dnmt complex to promote the CpG island methylation and reduce Dicer expression in ICC cells. CyclinD1 positively regulated the proliferation and invasion of ICC cells, which were mitigated by Dicer expression. The down-regulated Dicer expression decreased miR-1914-5p and miR-541-5p expression, which targeted CyclinD1 and CDK6 to form a feedback loop to promote the progression of ICC. Hence, our findings indicated that down-regulated Dicer expression was a biomarker for prognosis of ICC and suggest that CyclinD1 may be a therapeutic target for intervention of ICC.

## Conclusions

In conclusion, the findings uncover that CyclinD1 can enhance the Dicer promoter methylation to inhibit Dicer and relative miRNA expression, leading to progression of ICC.

## Additional files

Additional file 1: **Figure S1.** Dicer translocates to the nucleus in ICC cells. (A-C) Western blot analysis of Dicer localization of cytoplasm or nucleus in LV-siR-Dicer and control of ICC and HIBEpic cells. (D) Anti-HP1α immunoprecipitates endogenously SUV39H1, DNMT3A and DNMT3B from HuccT-1 and HCCC9810 cells. Data are representative images of each group from three separate experiments. (TIF 805 kb)

Additional file 2: **Figure S2.** Cyclin D1 in the transcriptional inhibition is general to the regulation of related downstream hypermethylated genes. (A-B) ChIP analysis of CyclinD1, HP1α, H3K9me3 and SUV39H1 enrichment in the SOX2 and HOXA2 promoter region. IgG served as a negative control. Relative enrichment fold = [%(ChIP/Input)]/[%(IgG/Input)]. *P < 0.05, **p < 0.01, ***p < 0.001. (TIF 1556 kb)

## Data Availability

The authors declare that all the data supporting the findings of this study are available within the article.

## Abbreviations

3’UTR: 3’untranslated region
BSP: Bisulfite sequencing PCR
CCA: Cholagiocarcinoma
CCK-8: Cell Counting Kit-8
ChIP: Chromatin immunoprecipitation
Co-IP: Co-immunoprecipitation
ECL: Enhanced chemiluminescence
FBS: Fetal bovine serum
HCC: Hepatocarcinoma
HP1α: Heterochromatin protein 1α
HRP: Horseradish peroxidase
ICC: Intrahepatic cholangiocarcinoma
IHC: Immunohistochemitry
OD: Optical density
PI: Propidium iodide
PVDF: Polyvinylidene fluoride
qRT-PCR: Quantitative real-time polymerase chain reaction
SDS-PAGE: Sodium dodecyl sulfate polyacrylamide gel electrophoresis
