# Supplementary figures and images for: CyclinD1 inhibits dicer and crucial miRNA expression by chromatin modification to promote the progression of intrahepatic cholangiocarcinoma
Source: J Exp Clin Cancer Res. 2019 Oct 7;38:413. doi: 10.1186/s13046-019-1415-5 (PMC6781400; doi:10.1186/s13046-019-1415-5)

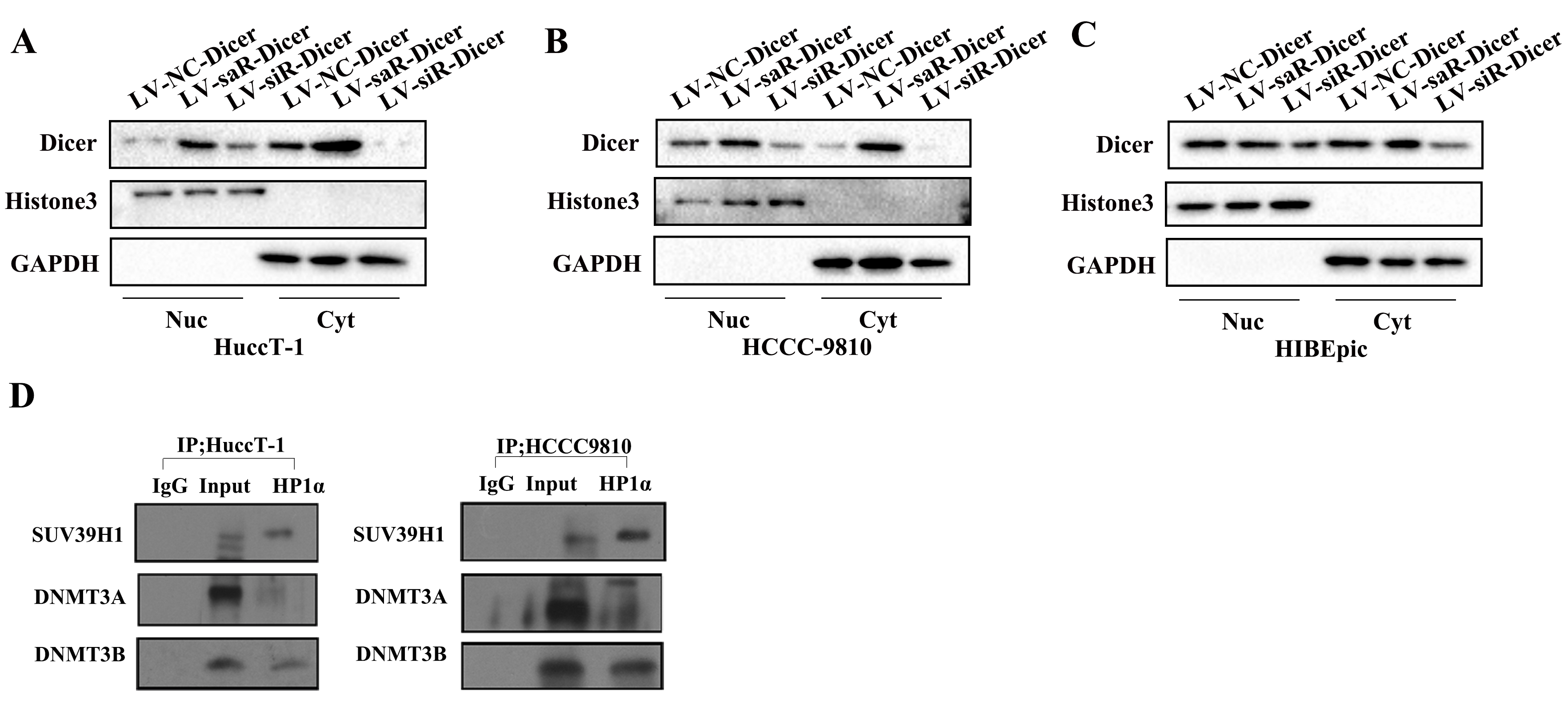

Supplement: Supplementary file 1 — Figure S1. Dicer translocates to the nucleus in ICC cells. (A-C) Western blot analysis of Dicer localization of cytoplasm or nucleus in LV-siR-Dicer and control of ICC and HIBEpic cells. (D) Anti-HP1α immunoprecipitates endogenously SUV39H1, DNMT3A and DNMT3B from HuccT-1 and HCCC9810 cells. Data are representative images of each group from three separate experiments. (TIF 805 kb) [file 13046_2019_1415_MOESM1_ESM.tif]

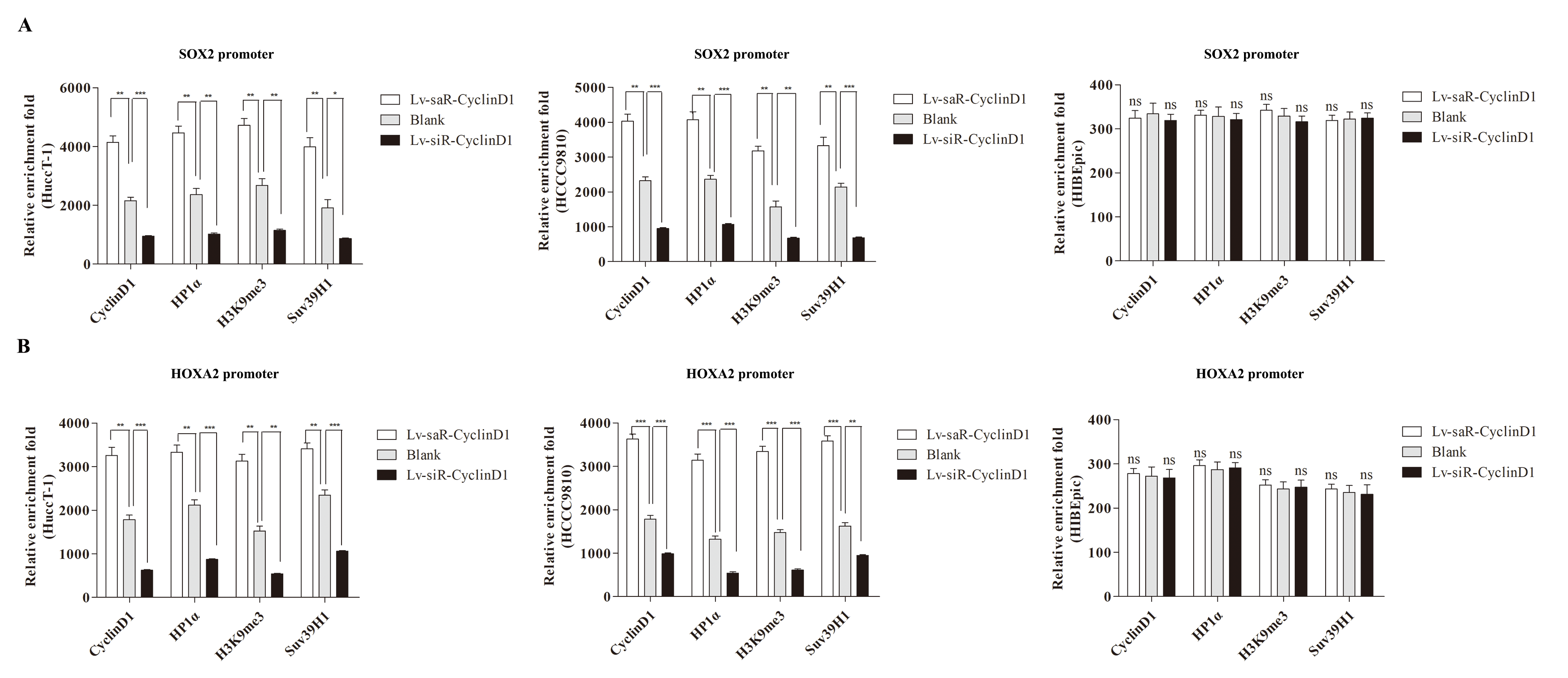

Supplement: Supplementary file 2 — Figure S2. Cyclin D1 in the transcriptional inhibition is general to the regulation of related downstream hypermethylated genes. (A-B) ChIP analysis of CyclinD1, HP1α, H3K9me3 and SUV39H1 enrichment in the SOX2 and HOXA2 promoter region. IgG served as a negative control. Relative enrichment fold = [%(ChIP/Input)]/[%(IgG/Input)]. *P < 0.05, **p < 0.01, ***p < 0.001. (TIF 1556 kb) [file 13046_2019_1415_MOESM2_ESM.tif]
